# Supplement of 5-hydroxytryptophan before induction suppresses inflammation and collagen-induced arthritis

**DOI:** 10.1186/s13075-015-0884-y

**Published:** 2015-12-15

**Authors:** Tao-Hsiang Yang, Peng-Yang Hsu, Menghsiao Meng, Che-Chun Su

**Affiliations:** Graduate Institute of Biotechnology, National Chung Hsing University, Taichung, Taiwan; College of Biotechnology and Bioresources, Da-Yeh University, Changhua, Taiwan; Department of Internal Medicine, Changhua Christian Hospital, 135, Nan-Hsiao Street, Changhua, Taiwan 500 ROC; Graduate Institute of Statistics and Information Science, National Changhua University of Education, Changhua, Taiwan; Department of Bioindustry Technology, Da-Yeh University, Datsuen, Changhua, Taiwan

**Keywords:** Rheumatoid arthritis, collagen-induced-arthritis, 5-Hydroxytryptophan, Inflammation, Suppression

## Abstract

**Background:**

Evidence is accumulating that a preclinical phase is present before the onset of clinical signs and symptoms of rheumatoid arthritis (RA). This phase represents an important therapeutic window within which interventions can dramatically modulate outcomes. An agent able to prevent RA for high risk individuals in this phase is therefore desired. In this study, we investigated whether tryptophan metabolite, 5-hydroxytryptophan (5-HTP) or 5-methoxytryptophan (5-MTP), can act as such an agent for primary prevention of collagen-induced arthritis (CIA).

**Methods:**

Mouse splenocytes were pretreated with 5-HTP or 5-MTP and activated by anti-CD3 plus anti-CD28 antibodies in vitro. The percentages of interferon-γ (IFNγ)^+^CD4^+^ T cells and interleukin-17 (IL-17)^+^CD4^+^ T cells were measured by flow cytometry. The production of pro-inflammatory cytokines, serotonin and kynurenine was measured by enzyme-linked immunosorbent assay. A CIA model was used to investigate the in vivo effects of 5-HTP on the prevention of arthritis.

**Results:**

5-HTP decreased the percentages of IFNγ^+^CD4^+^ T cells and IL-17^+^CD4^+^ T cells and suppressed the production of IL-2, IL-4, IL-6, IL-17, tumor necrosis factor-α (TNFα) and IFNγ in activated splenocytes. 5-HTP administered before induction decreased the disease activities in CIA mice and suppressed the production of TNFα, IL-6 and cyclooxygenase-2 in arthritic joints. 5-HTP also increased serotonin, but decreased kynurenine in the CIA mice.

**Conclusions:**

5-HTP suppresses inflammation and arthritis through decreasing the production of pro-inflammatory mediators. 5-HTP supplement before induction ameliorates arthritis in a CIA model.

## Background

Rheumatoid arthritis (RA) is a debilitating disease resulting in joint inflammation and deformity, leading to loss of daily functions. With the advent of biologic agents, clinical remission is achievable for many patients [1]. A new frontier in the research into RA is at the earliest stages of the disease. Discovery of circulating autoantibodies, specifically anti-citrullinated protein antibodies (ACPA), which are present years before clinical symptoms appear, helps to predict the chance of developing RA in the future [2–6]. Evidence is accumulating that a preclinical phase is present before onset of clinical signs and symptoms, and this phase represents an important therapeutic window within which interventions can dramatically modulate outcomes [2–6].

In addition to ACPA, smoking, genetic factors (HLA-DRB1 shared epitope), and family history also play a role in determining which individuals will ultimately develop arthritis [2]. Individuals at risk should stop smoking. However, we lack agents which can effectively reduce their risk. Because there are no symptoms or signs, an ideal agent for the primary prevention would entail efficacy, low cost and minimal side effects.

5-hydroxytryptophan (5-HTP) is an intermediate in serotonin synthesis. It can easily cross the blood–brain barrier and increase the serotonin levels in the central nervous system. Early studies found that 5-HTP monotherapy or 5-HTP used with other antidepressants can improve mood and depression. In certain countries, 5-HTP is used as a dietary supplement to treat depression, suppress appetite, and ameliorate insomnia [7, 8]. Recently, several studies found that 5-HTP is a potent mediator against inflammation. 5-HTP was found to suppress the activation of p38 and nuclear factor-κB (NF-κB) in fibroblasts [9], and decrease the expression of interleukin (IL)-17 in peripheral blood mononuclear cells (PBMCs) [10]. Moreover, 5-HTP suppresses the production of tumor necrosis factor-α (TNFα) in lipopolysaccharide (LPS)-treated mice [11] and improves allergic lung inflammation in a murine asthma model [12]. In addition, 5-HTP and its metabolite 5-methoxytryptophan (5-MTP) decreases the expression of cyclooxygenase-2 (COX-2) in A549 cells and suppresses cancer growth and metastasis in mice [13]. These findings prompted us to test whether 5-HTP can be an option for prevention or treatment of collagen-induced arthritis (CIA), an animal model for human RA.

We found that 5-HTP suppressed T cell activation and pro-inflammatory cytokine production in activated splenocytes. 5-HTP given before induction decreased the disease activities in CIA mice. Furthermore, 5-HTP increased the serotonin levels, but decreased the kynurenine levels in the CIA mice. These findings suggest that 5-HTP is a good agent for the prevention of CIA. Further studies are warranted to investigate whether 5-HTP prevents RA in healthy high-risk individuals in the preclinical phase.

## Methods

### Induction of CIA

DBA/1 mice were purchased from the Jackson Laboratory (Bar Harbor, ME, USA), and used at age 12–16 weeks under a protocol approved by the Institutional Animal Care and Use Committee at the Changhua Christian Hospital. For the induction of CIA, bovine type II collagen (2 mg/ml in 0.05 M acetic acid) was emulsified in complete Freund’s adjuvant (CFA) (containing 5 mg/ml of killed *Mycobacterium tuberculosis* H37Ra) at 4 °C for 12 hours. DBA/1 mice were injected intradermally at the base of the tail with 100 μg collagen in adjuvant at day 0, and boosted with an intraperitoneal (i.p.) injection of 100 μg collagen at day 21. The arthritis scores were monitored every other day following the booster immunization. Collagen and adjuvant were purchased from Chondrex, Inc. (Redmond, WA, USA).

CIA symptoms were evaluated visually in each limb and graded on a scale of 0–4 as follows: grade 0, no erythema or swelling; grade 1, erythema and swelling of the ankle joint; grade 2, mild erythema and mild swelling involving the entire paw; grade 3, moderate erythema and moderate swelling involving the entire paw; and grade 4, severe erythema and severe swelling involving the entire paw. The CIA score for each mouse was the sum of the scores for all four limbs (maximum score 16) [14].

### Histology scores for ankle joints

Ankle joints were fixed in 10 % paraformaldehyde. Following decalcification (Shandon TBD-2 decalcifier, Thermo Scientific, Waltham, MA, USA), dehydration and paraffin-embedding, ankle tissues were cut into 6-μm sections and stained with hematoxylin and eosin. The levels of synovial hyperplasia, cartilage erosion, and leukocyte infiltration were evaluated and scored separately on a scale of 0–2 points for each item (grade 0, normal; grade 1, mild; and grade 2, severe) [15].

### Administration of 5-HTP

The protocol is shown in Fig. 3a. Each group contained eight mice. DBA/1 mice were divided into six groups: group 1, treated with normal saline; groups 2 and 3: treated with 5-HTP (Sigma-Aldrich, St. Louis, MO, USA) at 32 and 160 mg/L, respectively, in drinking water, from day 7 before induction to day 35; groups 4 and 5: treated with 5-HTP at 32 and 160 mg/L, respectively, in drinking water, from day 20 to day 35; group 6, treated with 5-HTP at 4 mg/kg through i.p. injection every 3 days from day 7 before induction to day 35. The study was done twice with similar results.

### Immunohistochemistry (IHC) analysis of ankle joints

Ankle sections were processed through deparaffinization, rehydration and antigen retrieval, then incubated with rabbit anti-TNFα polyclonal antibodies (Abs) (Bioss, Woburn, MA, USA), anti-COX-2 Abs (clone: SP21, Thermo Scientific) or rabbit anti-IL-6 polyclonal Abs (Abcam, Cambridge, MA, USA) for 12 hours at 4 °C. Following incubation with goat anti-rabbit IgG detection system (GBI Labs., Mukilteo, WA, USA), 3,3-diaminobenzidine (DAB) Quanto substrate (Lab Vision Co., Freemont, CA, USA) was used for color development. Hematoxylin was used for counterstaining. The expression of cyclooxygenase-2 (COX-2), TNFα and IL-6 in the areas with mononuclear cell infiltration (positive area) were measured using a digital camera and Image-Pro Plus 6.0 software.

### Quantitative real-time polymerase chain reaction (PCR) for mRNA levels of pro-inflammatory proteins

Formalin-fixed, paraffin-embedded ankle joint sections were deparaffinized, rehydrated for 30 minutes at 37 °C, then digested by proteinase K (60 mg/ml, Amresco Inc., Solon, Ohio, USA) at 60 °C. Total RNA was extracted using a Quick-RNA™ MiniPrep kit (Zymo Research, Inc., Irvine, CA, USA), and complementary DNA (cDNA) was synthesized using a PrimeScript™ RT reagent kit (Takara Bio Inc., Kyoto, Japan). The SYBR Green-based real-time PCR technique (KAPA Biosystems Inc., Wilmington, MA, USA) was used to detect the messenger RNA (mRNA) levels of COX-2, TNFα, IL-6, interferon-γ (IFNγ), IL-22 and NF-κB (p65). The reaction was conducted with SYBG FAST qPCR master mix for 40 cycles at 60 °C on the ABI 7300 real-time PCR system (Applied Biosystems, Foster City, CA, USA). No template control was used for exclusion of contamination in the assay. The threshold cycle of each gene was normalized by β-actin expression and the final difference for each group was presented as n-fold changes over the control group.

In other experiments, splenocytes were treated with 5-HTP and stimulated with anti-CD3 (0.5 μg/ml) plus anti-CD28 (0.25 μg/ml) Abs for 12 hours. Antibodies were purchased from BD Biosciences (San Diego, CA, USA). Following total RNA isolation and cDNA synthesis, SYBR Green-based real-time PCR and the ABI 7300 real-time PCR system were used to detect the mRNA levels of COX-2, TNFα, IFNγ, IL-17, T-bet and RORγt.

### Analysis of IFNγ^+^CD4^+^ T cells and IL-17^+^CD4^+^ T cells

Splenocytes from DBA/1 mice were treated with 5-HTP or 5-MTP (Sigma-Aldrich) and stimulated with anti-CD3 plus anti-CD28 Abs for 36 hours. Protein transport inhibitors were added in the last 4 hours. Following fixation and permeabilization, splenocytes were incubated with fluorescein isothiocyanate (FITC)-conjugated anti-CD3 Abs (clone: 145-2C11), PE-Cy5-conjugated anti-CD4 Abs (clone: RM4-5) and phycoerythrin (PE)-conjugated anti-IFN-γ Abs (clone: XMG1.2) for 15 minutes. Standard 5-color flow cytometry (Beckman Coulter, FC-500) was used to measure the percentage of IFNγ^+^CD4^+^ T cells (IFNγ^+^CD3^+^CD4^+^/CD3^+^CD4^+^ cells).

For analysis of IL-17^+^CD4^+^ T cells, splenocytes treated with 5-HTP or 5-MTP were incubated with IL-6 (20 ng/ml), transforming growth factor-β (1 ng/ml), IL-23 (5 ng/ml), anti-mouse IL-4 Abs (1 μg/ml), anti-mouse IFN-γ Abs (1 μg/ml), anti-CD3 Abs and anti-CD28 Abs for 48 hours. Protein transport inhibitors were added in the last 4 hours. Following fixation and permeabilization, splenocytes were incubated with FITC-conjugated anti-CD3 Abs (clone: 145-2C11), PE-Cy5-conjugated anti-mouse CD4 Abs (clone: RM4-5) and PE-conjugated anti-mouse IL-17 Abs (clone: TC11-18H10) for 15 minutes. Flow cytometry was used to analyze the percentage of IL-17^+^CD4^+^ T cells (IL-17^+^CD3^+^CD4^+^/CD3^+^CD4^+^ cells). The antibodies were purchased from BD Biosciences, and recombinant proteins were purchased from Biolegend (San Diego, CA, USA).

In other experiments, splenocytes and lymph node cells from the CIA mice at day 36 post-immunization were stimulated with phorbol 12-myristate 13-acetate (PMA, 50 ng/ml, Calbiochem, San Diego, CA, USA) and ionomycin (500 ng/ml, Calbiochem) for 8 hours. Protein transport inhibitors (BD Biosciences) were added in the last 4 hours. Following fixation and permeabilization (BD Cytofix/Cytoperm™ kit), these cells were incubated with FITC-conjugated anti-CD3 Abs (clone: 145-2C11), PE-Cy5-conjugated anti-CD4 Abs (clone: RM4-5) and PE-conjugated anti-IFN-γ Abs (clone: XMG1.2) for 15 minutes. Antibodies were purchased from BD Biosciences. Flow cytometry was used to measure the percentage of IFNγ^+^CD4^+^ T cells.

### Analysis of cytokines

Serum samples were collected from CIA mice at day 36 post-immunization and the concentrations of IL-6, IL-17, TNFα and IFNγ were measured by cytometric bead array kits (BD Biosciences). In other experiments, splenocytes from DBA/1 mice were treated with 5-HTP in the presence of kynurenine (20 μg/ml, Sigma-Aldrich), fluoxetine (5 μg/ml, Sigma-Aldrich) or NSD-1015 (8.4 μg/ml, Sigma-Aldrich) and stimulated with anti-CD3 plus anti-CD28 Abs for 36 hours. The levels of TNFα (eBioscience, San Diego, CA, USA), IL-17 (R&D Systems, Minneapolis, MN, USA), IFNγ (eBioscience) and IL-2 (eBioscience) in the culture supernatants were measured by enzyme-linked immunosorbent assay (ELISA).

### Analysis of cell proliferation

Splenocytes from DBA/1 mice were treated with 5-HTP and stimulated with anti-CD3 plus anti-CD28 Abs for 18 or 36 hours. WST-1 (Clontech, Mountain View, CA, USA) was added in the last 2 hours and the absorbance was measured at 450 nm (reference wavelength 690 nm) by a Multiskan™ spectrophotometer (Thermo).

### Analysis of cell death

Splenocytes from DBA/1 mice were treated with 5-HTP and stimulated with anti-CD3 plus anti-CD28 Abs for 36 hours. The cells were incubated with 7-aminoactinomycin D (7-AAD) and FITC-conjugated annexin-V for 15 minutes. The percentage of cell death (7-AAD^+^Annexin-V^+^ cells/total cells) was measured by flow cytometry. All reagents were purchased from BD Biosciences. In other experiments, 5-HTP-treated splenocytes were processed through fixation and permeabilization, incubated with FITC-conjugated anti-active caspase-3 Abs (clone: C92-605, BD Biosciences) and PE-Cy5-conjugated anti-CD4 Abs (clone: RM4-5) for 15 minutes. Flow cytometry was used to measure the percentage of caspase-3^+^CD4^+^ cells (caspase-3^+^CD4^+^/CD4^+^ cells).

### Analysis of serotonin and kynurenine

Serum samples were collected from CIA mice in group 1, 3 and 6 of the two independent animal studies at day 36 post-immunization and the levels of serotonin and kynurenine were measured using a serotonin ELISA (Enzo Life Sciences Inc, Ann Arbor, MI, USA) and a KYN ELISA (Cusabio Biotech Co., Ltd., Wuhan Hubei, China).

### Analysis of PGE_2_ production

Serum samples were collected from CIA mice at day 36 post-immunization and the concentrations of prostaglandin E_2_ (PGE_2_) were measured by ELISA (Enzo). In other experiments, splenocytes from DBA/1 mice were treated with 5-HTP and stimulated with anti-CD3 plus anti-CD28 Abs for 36 hours. The levels of PGE_2_ in the culture supernatants were measured by ELISA.

### Western blotting

5-HTP-treated splenocytes were incubated with radioimmunoprecipitation assay (RIPA) lysis buffer (Millipore, Temecula, CA, USA) for 30 minutes. Whole cell lysates were quantified using a Bicinchoninic Acid Kit for protein determination (Sigma-Aldrich). The protein levels of COX-2 (Abcam), PGE_2_ receptor EP2 (Abcam), T-bet (Millipore) and RORγt (BD biosciences) were analyzed by western blotting and quantified by Kodak Image 1D software.

### Statistics

Statistical analysis was performed using SPSS v12.0 software. Multiple comparisons were performed using the Kruskal-Wallis test, if significant (*p* <0.05), then the Duncan test was used to find out which pairs were different. Spearman’s rank correlation was used to analyze the correlation between the levels of serotonin, kynurenine and arthritis scores. In addition, the Mann–Whitney test was used to analyze the effects of kynurenine, fluoxetine and NSD-1015 on the cytokine production in 5-HTP-treated splenocytes.

## Results

### 5-HTP inhibited cell proliferation and cytokine production in activated splenocytes

**Fig. 1:**
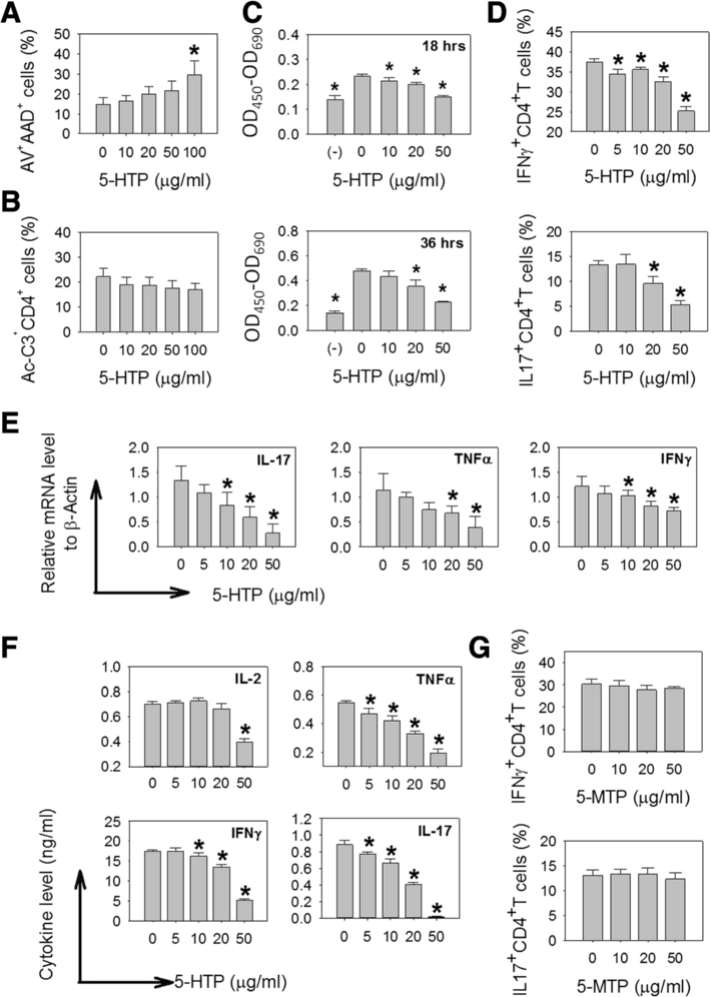
Effects of 5-hydroxytryptophan (*5-HTP*) on the activation of splenocytes. Splenocytes were treated with 5-HTP and stimulated with anti-CD3 plus anti-CD28 Abs. **a** Percentages of annexin V^+^ 7-aminoactinomycin D (*7-AAD*)^+^ cells (annexin V^+^ 7-AAD^+^ cells/total cells, mean ± SD). **b** Percentages of active caspase-3^+^CD4^+^ cells (active caspase-3^+^CD4^+^ cells/CD4^+^ cells, mean ± SD). **c**, WST-1 assay. Cell proliferation (mean ± SD) was defined as optical density (OD)_450_–OD_690_. (–): cells without activation. **d** Percentages (mean ± SD) of IFNγ^+^CD4^+^ T cells (IFNγ^+^CD4^+^CD3^+^ cells/CD4^+^CD3^+^ cells) and IL-17^+^CD4^+^ T cells (IL-17^+^CD4^+^CD3^+^ cells/CD4^+^CD3^+^ cells). **e** mRNA levels (mean ± SD) of IL-17, TNFα and IFNγ. **f** Protein levels (mean ± SD) of IL-2, IL-17, TNFα and IFNγ in culture supernatant. **g** Percentages of IFNγ^+^CD4^+^ T cells and IL-17^+^CD4^+^ T cells (mean ± SD). *Significant compared with no 5-HTP treatment. Multiple comparisons were performed using the Kruskal–Wallis test, if significant (*p* <0.05), then the Duncan test was used to find out which pairs were different

First, we analyzed the toxicity of 5-HTP to activated splenocytes and found that treatment of 5-HTP at a level no more than 50 μg/ml did not increase the levels of cell death, as compared with cells without 5-HTP treatment (Fig. 1a, b).

**Fig. 2:**
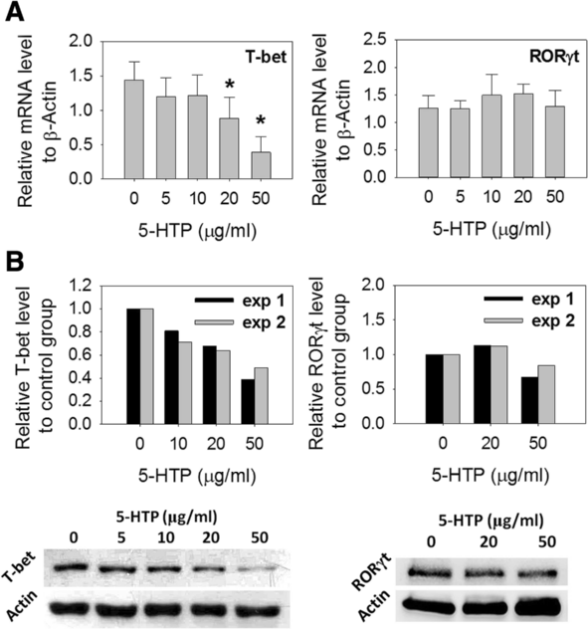
Effects of 5-hydroxytryptophan (*5-HTP*) on the expression of T-bet and RORγt in activated splenocytes. Splenocytes were treated with 5-HTP and stimulated with anti-CD3 plus anti-CD28 Abs. **a** mRNA levels (mean ± SD) of T-bet and RORγt. **b** Western blotting for T-bet and RORγt. *exp 1* and *exp 2* experiment 1 and 2. *Significant compared with no 5-HTP treatment. Multiple comparisons were performed using the Kruskal–Wallis test, if significant (*p* <0.05), then the Duncan test was used to find out which pairs were different

Next, we studied the effects of 5-HTP on the activation of splenocytes. The absorbance (optical density (OD)_450_–OD690) in WST-1 treated cells is an index of cell proliferation [16]. The cell activity in activated splenocytes was decreased with 5-HTP at 10, 20 or 50 μg/ml (Fig. 1c). At these levels, 5-HTP decreased the percentages of IFNγ^+^CD4^+^ T cells and IL-17^+^CD4^+^ T cells in activated splenocytes (Fig. 1d), and significantly decreased the levels of mRNA and protein for IFNγ, TNFα and IL-17 (Fig. 1e, f). In contrast, IL-2 production was decreased only at a level of 50 μg/ml. Furthermore, 5-HTP decreased the mRNA levels of T-bet, but did not inhibit RORγt expression (Fig. 2a). These findings suggest that 5-HTP regulates cell proliferation and inhibits production of pro-inflammatory cytokines in activated splenocytes.

We also studied the effects of 5-MTP, a metabolite of 5-HTP, on the activation of splenocytes and found that 5-MTP given at 10, 20 or 50 μg/ml did not affect the percentages of IFNγ^+^CD4^+^ T cells or IL-17^+^CD4^+^ T cells (Fig. 1g).

### Supplement of 5-HTP before induction decreased disease activities in CIA mice

**Fig. 3:**
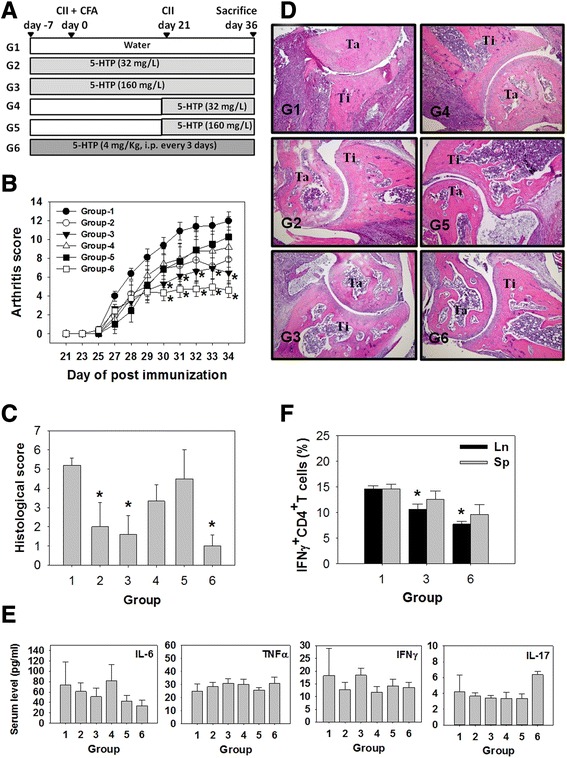
Effects of 5-hydroxytryptophan (*5-HTP*) on disease activity in the mice with collagen-induced arthritis (CIA). **a** Protocol for CIA induction and 5-HTP supplement. *CII* bovine type II collagen, *CFA* complete Freund’s adjuvant. **b** and **c** Arthritis scores and histological scores (mean ± standard error (SE)). **d** Histological staining of arthritic ankle joints. *Ti* tibia, *Ta* talus. **e** Serum cytokine levels (mean ± SE). **f** Percentages of IFNγ^+^CD4^+^ T cells (IFNγ^+^CD4^+^CD3^+^ cells/CD4^+^CD3^+^ cells, mean ± SE) in the spleen (*Sp*) and inguinal lymph nodes (*Ln*). *Significant compared with group 1. Each group contained eight mice. Multiple comparisons were performed using the Kruskal–Wallis test, if significant (*p* <0.05), then the Duncan test was used to find out which pairs were different

We investigated the effects of 5-HTP on the development of CIA. The protocol for CIA induction and 5-HTP supplementation is presented in Fig. 3a. 5-HTP did not affect the body weight of these mice. We did not observe any side effects in mice receiving oral 5-HTP (groups 1–5); however, parenteral 5-HTP induced a bout of mild diarrhea within 30 minutes after injection (group 6). The average consumption of water in each mouse was 5 ml per day, thus the daily consumption of 5-HTP was equivalent to 384 mg (groups 2 and 4) and 1,920 mg (groups 3 and 5) for a 60-kg person.

We found that the arthritis scores in group 3 and group 6 were significantly lower than those in group 1 (Fig. 3b). Histology scores were also decreased in group 3 and group 6 (Fig. 3c, d). These results suggest that 5-HTP given before induction reduces CIA activity.

### 5-HTP decreased the production of TNFα and IL-6 in arthritic joints

**Fig. 4:**
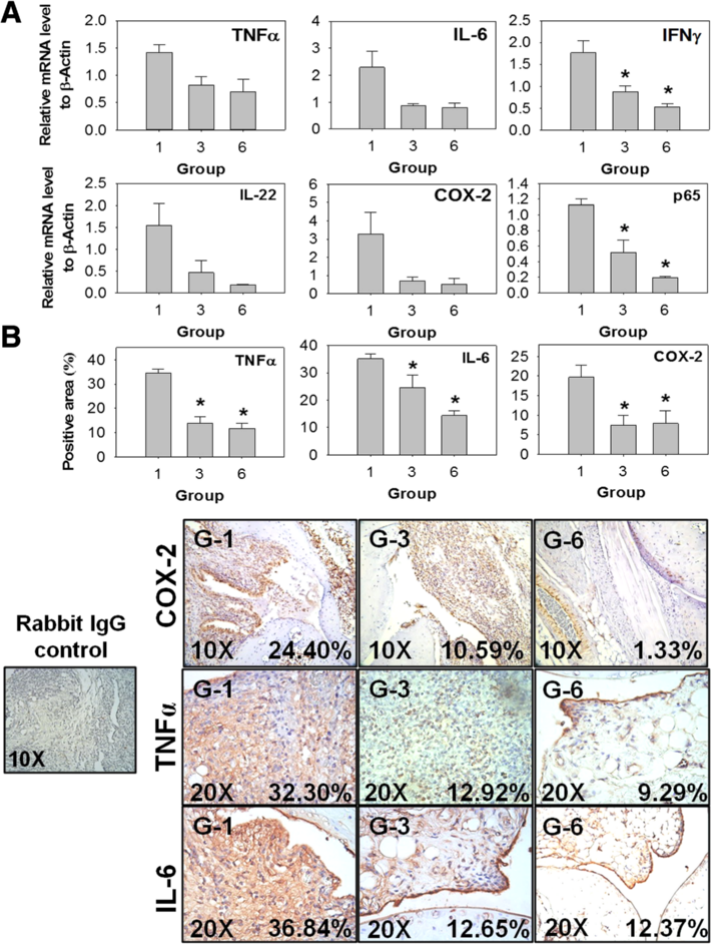
Effects of 5-hydroxytryptophan (*5-HTP*) on the expression of pro-inflammatory mediators in the arthritic joints. **a** mRNA levels (mean ± standard error) of TNFα, IL-6, IFNγ, IL-22, cyclooxygenase-2 (*COX-2*) and NF-κB (p65) in the arthritic joints. **b** Immunohistochemistry staining for TNFα, IL-6 and COX-2 in the arthritic ankles. The expressions of these proteins in the areas with mononuclear cell infiltration (positive area) were measured using a digital camera and Image-Pro Plus 6.0 software (Media Cybernetics Inc., Bethesda, MD, USA). The grouping was as described in Fig. 3a. At least four mice in each group were studied. *Significant compared with group 1. Multiple comparisons were performed using the Kruskal–Wallis test, if significant (*p* <0.05), then the Duncan test was used to find out which pairs were different

We investigated whether 5-HTP suppresses arthritis through decreasing the production of pro-inflammatory cytokines in arthritic joints. As shown in Fig. 4a, CIA mice pretreated with 5-HTP (groups 3 and 6) had lower mRNA levels of IFNγ and NF-κB (p65) compared with control mice (group 1). 5-HTP also decreased the mRNA levels of TNFα, IL-6 and IL-22; however, the differences were not statistically significant (Fig. 4a). Furthermore, immunohistochemistry analysis showed that 5-HTP decreased the production of TNFα and IL-6 in the arthritic ankles (Fig. 4b).

We next studied the levels of cytokines in the serum and found that 5-HTP did not affect the levels of IL-6, TNFα, IFNγ and IL-17 (Fig. 3e). In addition, 5-HTP decreased the percentage of IFNγ^+^CD4^+^ T cells in the inguinal lymph nodes, but not in the spleen (Fig. 3f).

### 5-HTP decreased the production of COX-2 and PGE_2_ in activated splenocytes and in CIA mice

**Fig. 5:**
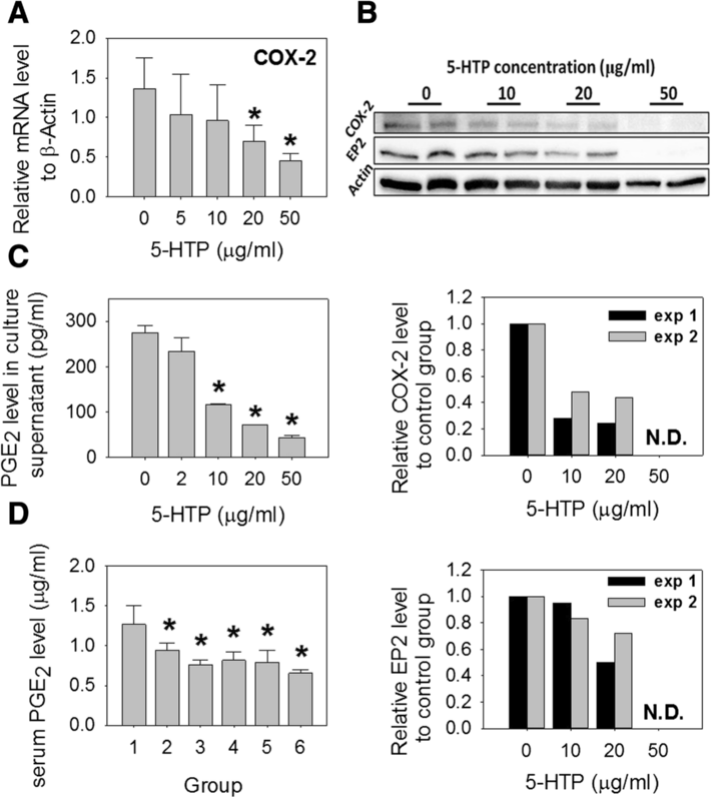
Effects of 5-hydroxytryptophan (*5-HTP*) on the expression of cyclooxygenase-2) (*COX-2* and prostaglandin E_2_ (*PGE*_*2*_) in activated splenocytes and mice with collagen-induced arthritis (CIA). **a** mRNA levels (mean ± SD) of COX-2 in activated splenocytes. **b** Protein levels of COX-2 and PGE_2_ receptor EP-2 in activated splenocytes. *N.D.* undetectable. *exp 1* and *exp 2* experiment 1 and 2. **c** and **d** PGE_2_ levels (mean ± standard error) in the culture supernatants and mice serum. *Significant compared with no 5-HTP treatment cells (**a** and **c**) or group 1 (**d**). The grouping (**d**) was as described in Fig. 3a. At least six mice in each group were studied (**d**). Multiple comparisons were performed using the Kruskal–Wallis test, if significant (*p* <0.05), then the Duncan test was used to find out which pairs were different

5-HTP suppressed the expression of COX-2 and PGE_2_ receptor EP2 in activated splenocytes and reduced the PGE_2_ levels in culture supernatants (Fig. 5a–c). In CIA mice, 5-HTP also decreased the serum levels of PGE_2_ (Fig. 5d) and suppressed the expression of COX-2 in inflamed ankle joints (Fig. 4).

### 5-HTP given orally increased serotonin but decreased kynurenine in the serum of CIA mice

**Fig. 6:**
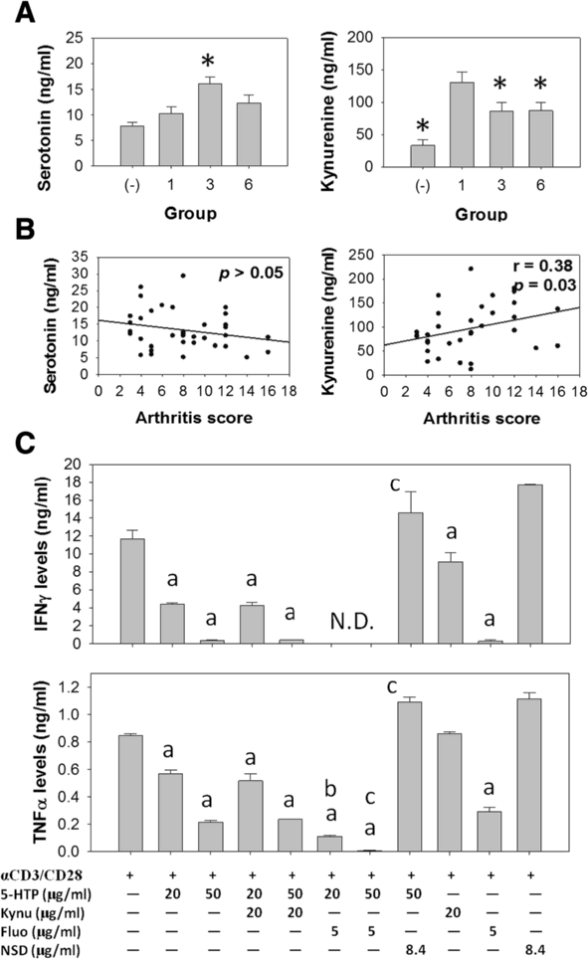
Effects of 5-hydroxytryptophan (5-HTP) on the production of serotonin and kynurenine in the mice with collagen-induced arthritis (CIA). Serum levels of serotonin and kynurenine in the CIA mice from groups 1, 3 and 6 were measured by ELISA. **a** Serum levels of serotonin and kynurenine (mean ± standard error) in the CIA mice. *Significant compared with group 1. *(–)* same-aged DBA/1 mice. The grouping was as described in Fig. 3a. Multiple comparisons were performed using the Kruskal–Wallis test, if significant (*p* <0.05), then the Duncan test was used to find out which pairs were different. **b** Correlations between the levels of serotonin, kynurenine and arthritis scores in the CIA mice. Statistical analysis was performed using Spearman’s rank correlation. **c** Splenocytes were treated with 5-HTP alone or in combination with kynurenine, fluoxetine or NSD-1015, and then stimulated with anti-CD3 plus anti-CD28 Abs. Cytokine levels (mean ± SD) of TNFα and IFNγ in culture supernatants were measured *N.D.* undetectable. *a* Significant compared with control group/column one. *b* Significant compared with 5-HTP (20 μg/ml). *c* Significant compared with 5-HTP (50 μg/ml). The Mann–Whitney test was used to analyze the effects of kynurenine (*Kynu*), fluoxetine (*Fluo*) and NSD-1015 on the cytokine production in 5-HTP-treated splenocytes

To analyze the effects of 5-HTP supplement on tryptophan metabolism in CIA mice, serum levels of serotonin and kynurenine from CIA mice in groups 1, 3 and 6 were measured by ELISA. We found that CIA *per se* markedly increased kynurenine levels in the mice (control group with no induction, 33.50 ± 9.10 ng/ml; group 1, 138.86 ± 15.71 ng/ml; mean ± SE); however, 5-HTP attenuated this effect (group 3, 80.40 ± 12.41 ng/ml; group 6, 80.02 ± 10.92 ng/ml; mean ± SE) (Fig. 6a). In contrast, CIA did not affect the serotonin levels in the mice (control group with no induction, 7.85 ± 0.76 ng/ml; group 1, 10.29 ± 1.28 ng/ml; mean ± SE); however, 5-HTP taken orally significantly increased the serotonin level (group 3, 16.18 ± 1.34 ng/ml; mean ± SE) (Fig. 6a). In addition, we analyzed the relationships between the serum levels of tryptophan metabolites and disease activity, and found that kynurenine levels were positively correlated with arthritis scores (*r* = 0.38, *p* = 0.03) (Fig. 6b).

### Tryptophan metabolites and inflammation

Certain selective serotonin re-uptake inhibitors (SSRIs) can suppress the development of CIA in mice and decrease the production of pro-inflammatory cytokines in human synovial membrane cells. In this study, we found that fluoxetine suppressed the production of TNFα and IFNγ in activated splenocytes. Moreover, fluoxetine and 5-HTP further decreased the production of the cytokines (Fig. 6c). We also studied the effects of NSD-1015, an inhibitor of aromatic amino acid decarboxylase on the cytokine production and found that NSD-1015 increased the production of TNFα and IFNγ in activated splenocytes. NSD-1015 also restored the production of TNFα and IFNγ inhibited by 5-HTP (Fig. 6c). In addition, kynurenine decreased IFNγ production in activated splenocytes. In contrast, kynurenine did not affect the production of TNFα and IFNγ in 5-HTP-treated splenocytes (Fig. 6c). These results suggest that there are complicated interplays between tryptophan metabolites and inflammation.

## Discussion

Tryptophan is an essential amino acid for humans. It has two major metabolic pathways: the serotonin pathway and kynurenine pathway. In the serotonin pathway, tryptophan is catalyzed into 5-HTP by tryptophan hydroxylase-1 (TPH-1) and then converted into serotonin. In the kynurenine pathway, tryptophan is catalyzed into N-formylkynurenine by indoleamine 2,3 dioxygenase (IDO) and then converts into kynurenine. 5-HTP suppresses inflammatory responses in murine models of asthma and sepsis [11, 12]. 5-HTP also inhibits the production of pro-inflammatory mediators in different cell lines [9, 10, 13]. These findings made us interested in studying whether 5-HTP can suppress inflammation and disease activity in CIA mice.

We found that 5-HTP given at 10, 20 and 50 μg/ml suppressed cell proliferation and decreased the production of IFNγ^+^CD4^+^ T cells and IL-17^+^CD4^+^ T cells in activated splenocytes. 5-HTP also suppressed the expression of IL-17, TNFα, IFNγ and T-bet in activated splenocytes. These findings did not result from cell death, because we showed that 5-HTP did not increase cell death at these levels.

In animal studies, a supplement of 5-HTP from day 20 was not found to affect the disease course; however, 5-HTP given from day 7 before induction significantly decreased the arthritis scores and joint inflammation. These results suggest that 5-HTP supplement can be an option for prevention of arthritis.

5-HTP taken orally was shown to suppress allergic lung inflammation even though cytokine levels were not decreased on bronchoalveolar lavage [12]. In the current study, we also found that 5-HTP did not affect the cytokine levels in the serum and the percentages of IFNγ^+^CD4^+^ T cells in the spleen. However, 5-HTP suppressed the expression of TNFα and IL-6 in the inflamed ankle joints and decreased the percentages of IFNγ^+^CD4^+^ T cells in the draining lymph nodes. These results suggest that 5-HTP decreased arthritis activity without affecting systemic immunity.

Decreased levels of serotonin in the central nervous system are associated with major depressive disorders. Treatment with SSRIs or supplement of serotonin precursors is an important strategy in depression therapy. SSRIs can block serotonin re-uptake and thus increase serotonin levels in the brain and improve depression [17–19]. Interestingly, certain SSRIs can decrease the production of pro-inflammatory cytokines [20, 21], suppress airway inflammation in asthma patients [22], and reduce disease activity in RA patients [23]. SSRIs have also been found to decrease the arthritis scores in CIA mice and suppress cytokines production in macrophages and synovial membrane cells [24]. In this study, we found that fluoxetine effectively decreased the production of IFNγ and TNFα in activated splenocytes.

In our animal study we found that 5-HTP given orally increased the serum levels of serotonin, whereas parenteral 5-HTP did not affect the serum levels of serotonin in CIA mice. These results suggest that regulation of the serotonin levels is unlikely the major mechanism behind the suppression of arthritis by 5-HTP in the CIA mice.

RA patients have increased kynurenine levels in the blood [25–27] and the levels are positively correlated with C-reactive protein [28, 29]. In addition, RA patients have increased IDO activity in the synovial fluid [26]. Interestingly, pro-inflammatory cytokines such as TNFα, IL-1 and IFNγ can increase IDO expression and promote serotonin re-uptake, resulting in increased levels of kynurenine and decreased levels of serotonin [7, 30–33]. Our study showed that mice with a higher arthritis score were more likely to have high serum levels of kynurenine (Fig. 6b). Most of these mice received 5-HTP before or after immunization.

5-HTP is indicated for depression, obesity, headaches, fibromyalgia and insomnia. Administration of 5-HTP results in nausea, diarrhea, and vomiting in some patients; nevertheless, these side effects are mild and there are no significant laboratory abnormalities in these patients. In short, 5-HTP supplement is well-tolerated and causes minimal side effects. In clinical studies, the doses of 5-HTP in the treatment of depression have been from 20 to 3,250 mg per day [7, 8, 34, 35]. Treatment with 5-HTP at 600 mg per day was also found to decrease the frequency of migraine and improve insomnia [8]. In a murine model of asthma, the amount of 5-HTP given to the mice was equivalent to consumption of 200 mg per day by a 45-kg person [12]. In our animal study, 5-HTP given orally did not affect body weight or cause diarrhea. However, the daily consumption of 5-HTP was equivalent to 384 mg and 1,920 mg per day by a 60-kg person, respectively. Furthermore, 5-HTP given by i.p. injection at 30, 100 and 300 mg/kg was found to decrease the production of TNFα in a sepsis model [11]. Parenteral 5-HTP is known to increase intestinal peristalsis resulting in diarrhea. In order to avoid severe diarrhea and dehydration, we gave the mice 5-HTP by i.p. injection at a much lower dose (4 mg/kg every 3 days). Even though mild diarrhea still occurred after each injection, it was well-tolerated. More importantly, these mice had improved arthritis scores and decreased joint inflammation.

## Conclusions

This study provides in vitro and in vivo evidence that 5-HTP, a tryptophan metabolite, can regulate immune responses. 5-HTP supplement before induction can decrease disease activity, suppress joint inflammation and cause minimal side effects in CIA mice. Further studies are required to elucidate whether 5-HTP, a common dietary supplement can act as an agent for primary prevention of RA.

## Abbreviations

5-HTP: 5-hydroxytryptophan
5-MTP: 5-methoxytryptophan
7-AAD: 7-aminoactinomycin D
Abs: antibodies
ACPA: anti-citrullinated protein antibodies
CII: type II collagen
cDNA: complementary DNA
CFA: complete Freund’s adjuvant
CIA: collagen-induced arthritis
COX-2: cyclooxygenase-2
ELISA: enzyme-linked immunosorbent assay
FITC: fluorescein isothiocyanate
i.p. injection: intraperitoneal injection
IDO: indoleamine 2,3 dioxygenase
IFNγ: interferon-γ
IHC: immunohistochemistry
IL: interleukin
LPS: lipopolysaccharide
mRNA: messenger RNA
NF-κB: nuclear factor-κB
OD: optical density
PBMC: peripheral blood mononuclear cells
PE: phycoerythrin
PCR: polymerase chain reaction
PGE_2_: prostaglandin E_2_
PMA: phorbol 12-myristate 13-acetate
RA: rheumatoid arthritis
SSRI: selective serotonin re-uptake inhibitors
TNFα: tumor necrosis factor-α
TPH-1: tryptophan hydroxylase-1
